# Pills and ills: adverse events associated with oral antibiotics for prolonged durations of COpAT treatment courses

**DOI:** 10.1017/ash.2026.10373

**Published:** 2026-05-05

**Authors:** Nikhut Siddique, Russell J. Benefield, Heather Cummins, Laura K. Certain

**Affiliations:** 1 Internal Medicine- Division of Infectious Disease, University of Utah Health, USA; 2 Pharmacy, Pharmacotherapy, University of Utah Health, USA; 3 Internal Medicine, University of Utah Health, USA

## Abstract

**Background:**

Long-term oral antibiotic regimens are increasingly prescribed in place of outpatient parenteral antimicrobial therapy(OPAT). Many of these oral agents are used at higher doses and for longer durations than studied previously. The aim of this study was to describe the rates of adverse drug events (ADEs) among patients discharged on long-term oral antibiotic regimens at our center.

**Methods:**

This is a retrospective cohort study of adult patients discharged between January 1, 2021, and December 31, 2022, on oral antibiotic regimens of at least two weeks planned duration under the direction of an Infectious Diseases (ID) physician. The primary outcome was the occurrence of an ADE while on antibiotics up to ninety days after discharge.

**Results:**

Of 174 patients included, 46 (26%) experienced at least one ADE. Gastrointestinal ADE were most common, and were most frequently associated with linezolid (11.3 ADE/1,000 COpAT-days), rifampin (8.0 ADE/1,000 COpAT-days), and fluoroquinolones (5.4 ADE/1,000 COpAT-days). Other ADE included hyperkalemia and nephrotoxicity from trimethoprim-sulfamethoxazole (3.9 and 6.2 ADE/1,000 COpAT-days respectively), and bone marrow suppression from linezolid (7.5 ADE/1,000 COpAT-days). Linezolid (22.6 ADE/1,000 COpAT-days, 95% CI [9.81–35.39]) and trimethoprim-sulfamethoxazole (14.61 ADE/1,000 COpAT-days, [9.00–20.23]) were associated with the highest rates of ADE, while amoxicillin-clavulanate (3.28 ADE/1,000 COpAT-days, [0.66–5.91]), tetracyclines (3.28 ADE/1,000 COpAT-days, [0.07–6.50]), and amoxicillin (5.80 ADE/1,000 COpAT-days, [0.00–12.37]) had the lowest.

**Conclusion:**

Most patients tolerated oral therapy well with gastrointestinal adverse events being the most common ADE. Linezolid and trimethoprim-sulfamethoxazole were associated with the highest rates of adverse events.

## Introduction

Outpatient Parenteral Antimicrobial Therapy (OPAT) is the common practice of administering intravenous antibiotics in the community setting, and the risks associated with it are known.^1–3^ The landmark trials of Oral Versus Intravenous Antibiotics for Bone and Joint Infection (OVIVA) and Partial Oral versus Intravenous Antibiotic Treatment of Endocarditis (POET) demonstrated noninferiority with appropriately chosen oral antibiotics when compared to intravenous antibiotics for prolonged treatment courses.^4,5^ Other studies have evaluated clinical outcomes of patients discharged on prolonged courses of oral antibiotics compared to IV antibiotics, and generally suggest similar efficacy.^6,7^ These studies collectively have led to increased use of prolonged oral antibiotics as an alternative to OPAT in clinical practice for certain severe infections.^8^ The use of oral antibiotics for an extended period with laboratory monitoring in the outpatient setting is now known as “Complex Outpatient Antimicrobial Therapy” (COpAT).^8,9^

With the shift from OPAT to COpAT, there is a need for data on the relative safety and long-term effects of oral antibiotics used in this way. While COpAT avoids the risks inherent in long-term IV antibiotics (ie, line complications) patients may still experience side effects from long-term oral antibiotic therapy. Because many agents used as COpAT are used at higher doses or for longer durations than studied in registration trials, these side effects may be more common or more severe than with shorter durations of oral antibiotics. The actual risk of COpAT is not well established and the relative hazard between different COpAT agents is poorly described in current literature.^8,9^ A more comprehensive understanding of adverse events and rates associated with prolonged oral antibiotics will help guide appropriate monitoring for these patients. The objective of this study was to determine the rates of adverse drug events (ADEs) among patients discharged on COpAT regimens at our center.

## Methods

We conducted a retrospective cohort study of patients discharged from University of Utah Health (UUH) from January 1, 2021, to December 31, 2022. Patients were identified by searching the Enterprise Data Warehouse of UUH for inpatient encounters with a signed OPAT note for antibiotics by an Infectious Diseases (ID) consult service. (We currently do not use the term COpAT at our institution; patients discharged on prolonged oral antibiotics have an OPAT note.) Patients were included if they were at least 18 years of age and discharged on a planned course of at least two weeks of oral antibiotics between 1/1/21 and 12/31/22. If patients had more than one OPAT note due to multiple hospital admissions during the study period, only the first OPAT encounter was included in our study. Patients were excluded if they were: discharged on a combination of oral and intravenous antimicrobials (including antibiotic, antiviral, and antifungal); discharged on a course of antibiotics planned to last less than two weeks after discharge; followed by an outside infectious disease provider; discharged on oral antifungal or antiviral medication; treated for mycobacterial infections; had clear documentation of patient non-adherence; or discharged to a long-term acute care hospital, prison, or hospice. Since combination therapy is common, patients prescribed more than one oral antibiotic were included. An institutional guideline to guide oral antibiotic selection was available at our institution, but ultimately selection was based on the judgement of the treating ID provider.^10^ All patients had infectious disease clinic follow-up appointments scheduled.

### Outcomes

The primary outcome was the occurrence of ADE during the antibiotic course or up to ninety days after discharge, whichever occurred first. The occurrence of *Clostridioides difficile* infection was included as a secondary outcome.

We relied on clinician documentation to determine both the occurrence of ADE and the necessity of therapy modification based on the nature of the ADE. All included patient charts were reviewed by one individual to determine outcomes and adverse events based on clinician documentation. A second reviewer then conducted a secondary patient chart review to verify these findings. All instances of nephrotoxicity identified by the treating clinician met the Kidney Disease Improving Global Outcomes (KDIGO) AKI criteria: a ≥0.3 mg/dL increase in serum creatinine within 48 hours or a ≥1.5 times increase in serum creatinine from baseline within the past 7 days.^11^

For patients who experienced an ADE while receiving combination therapy, clinicians usually specified to which antibiotic the ADE was attributed. However, in cases where it was unknown, the ADE was ascribed to both drugs. If a single patient developed multiple ADEs, then each ADE was counted individually.

If an ADE occurred prior to the completion of a two-week course that required therapy cessation or change in antibiotic, these patients were still included. In addition, the drug they were changed to was also included in our analysis if the intended course was longer than two weeks.

### Statistical analysis

Descriptive data were summarized using counts and percentages or medians and interquartile ranges as appropriate. Adverse event rates were determined by dividing the number of adverse events observed by the number of days exposure to each antibiotic and reported as adverse events per 1,000 COpAT-days. Incidence rate differences in adverse events between antibiotic regimens with 95% CIs were calculated using the fmsb package in R version 4.4.1 (R Core Team 2024).^12^ ADE rates between comparator groups were considered statistically significant if the 95% CI for their difference did not contain zero.

The study protocol, including a waiver of informed consent, was approved by the UUH Institutional Review Board under Protocol 0011128.

## Results

**Figure 1.**
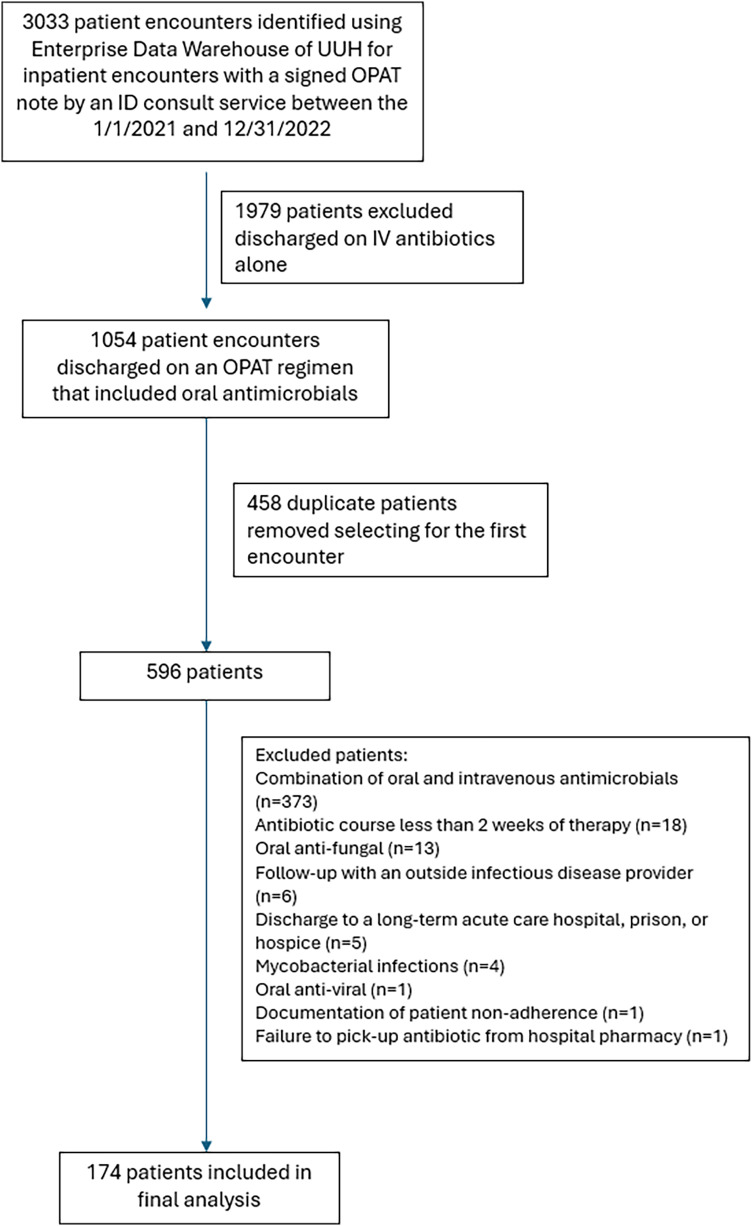
Exclusion criteria.

Of 3,033 patient encounters screened, 174 patients were included (Figure 1). The most common reasons for exclusion were patients discharged on a full or partial IV regimen, or the presence of duplicate patients due to a secondary OPAT encounter during the study period. Baseline characteristics of the included patients are summarized in Table 1. Bone and joint infections were the most common indication overall. Over one-third (38.5%) of patients were treated with combination regimens. The ADE patient population had a higher median age and higher percentage of immunosuppression and multi-drug antibiotic regimen compared to the total patient population. The most utilized antibiotics were amoxicillin-clavulanate, trimethoprim-sulfamethoxazole (TMP/SMX), and fluoroquinolones Overall, 8,590 patient-days of oral antibiotic therapy were observed.

**Table 1.** Baseline characteristics of study population

	Total patients (n = 174)	ADE patients (n = 46)
Sex, male^a^	121 (70)	30 (65)
Age, years	55 (41–65)	60 (48–66)
Race, Caucasian	139 (80)	37 (80)
Ethnicity, Hispanic/Latino	17 (10)	5 (11)
Co-morbidities		
Diabetes	58 (33)	15 (33)
Chronic kidney disease	9 (5)	3 (7)
Hemodialysis	1 (0.6)	1 (2)
Immunosuppression^b^	20 (11)	11 (24)
Primary language other than English	8 (5)	3 (7)
Currently experiencing homelessness	17 (10)	1 (2)
IV drug use history	25 (14)	1 (2)
Antibiotic duration, days	35 (25–42)	38 (22–47)
Multi-drug antibiotic regimen	67 (39)	24 (52)
Indication		
Bone or joint	107 (61)	34 (74)
Intra-abdominal/pelvic	17 (10)	5 (11)
Bacteremia/endocarditis	14 (8)	2 (4)
Central nervous system	10 (6)	1 (2)
Skin or soft tissue	6 (3)	0 (0)
Pulmonary	3 (2)	2 (4)
Genitourinary	2 (1)	0 (4)
Multisystem	15 (9)	2 (4)

a Data presented as n (%) or median (IQR) as appropriate.

b Defined as on active chemotherapy, on a biologic agent, or on corticosteroids.

Forty-six patients (26%) experienced 73 ADEs. Overall, gastrointestinal adverse events were the most common ADE (n = 37), followed by nephrotoxicity (11), hyperkalemia (8), bone marrow suppression (4), and rash (3). The number and characterization of ADEs found with each antibiotic class is listed in Supplementary Table 1. Forty-two patients required treatment modification in response to an ADE: either switching therapy (18), discontinuation/self-discontinuation of therapy (19), dose adjustment (2), or addition of an antiemetic (3). Four patients required admission related to an ADE (GI side effects [n = 2], nephrotoxicity and/or hyperkalemia [n = 2]).

**Figure 2.**
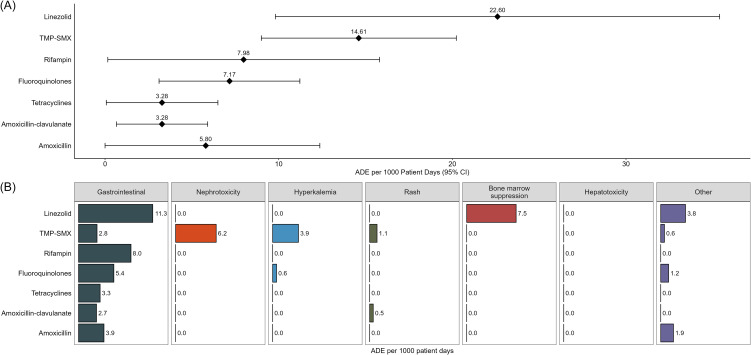
Adverse drug event rates. (A) Forest plot of point estimates with 95% CIs of adverse events per 1000 COpAT-days for the most utilized COpAT agents. (B) Split bar chart of adverse event rates by type of adverse event.

Figure 2 demonstrates the overall adverse drug event per 1,000 COpAT-days of all regimens for each antibiotic class. Linezolid and TMP/SMX had the highest rates of ADE while tetracyclines, amoxicillin-clavulanate, and amoxicillin had the lowest (Figure 2). Gastrointestinal adverse events were most frequently associated with linezolid, rifampin, and fluoroquinolones. No cases of *Clostridioides difficile* were reported. Other ADEs observed with some regularity included hyperkalemia and nephrotoxicity with TMP-SMX and bone marrow suppression with linezolid.

We further analyzed patients on monotherapy versus combination therapy (Table 2). Adverse events were more frequent in patients treated with combination regimens than monotherapy, but this was not statistically significant. (9.85 vs 6.98 ADE/1,000 COpAT-days, difference 2.87 ADE/1,000 COpAT-days, 95% CI [−1.22 to 6.97]). The most hazardous regimens observed included combination regimens with linezolid, TMP/SMX, or a fluoroquinolone, or monotherapy with linezolid or TMP/SMX. Tetracyclines and beta-lactams, either alone or in combination with other agents, were better tolerated.

**Table 2.** Adverse drug events (ADE) by regimen

Monotherapy regimens					
Drug	Number of treatment courses	Days of Therapy	Number of ADEs	ADE per 1,000 COpAT-days	ADE observed
Beta-lactam	43	1,456	7	4.81 (1.25–8.37)	Gastrointestinal: 6 Allergy (rash): 1
Fluoroquinolone^a^	18	774	2	2.58 (0.00–6.17)	Body aches: 1 Gastrointestinal: 1
Linezolid	10	246	4	16.26 (0.33–32.19)	Gastrointestinal: 2 Tremor: 1 Tongue soreness: 1
Doxycycline	11	364	2	5.49 (0.00–13.11)	Gastrointestinal: 2
TMP-SMX	40	1,170	13	11.11 (5.07–17.15)	Nephrotoxicity: 7 Hyperkalemia: 4 Allergy (rash): 1 Gastrointestinal: 1
**Total**	**122**	**4,010**	**28**	**6.98** **(4.40–9.57)**	Gastrointestinal: 13 Nephrotoxicity: 7 Hyperkalemia: 4 Allergy (rash): 1 Other: 3 (body aches, tremor)

Combination regimens^b^					
Beta-lactam + Other	32	1,094	4	3.66 (0.01–7.24)	Gastrointestinal: 2 Other: 2 (mouth sores, confusion)
Fluoroquinolone + Other	28	900	10	11.11 (4.22–18.00)	Gastrointestinal: 8 Hyperkalemia: 1 Joint pain:1
Linezolid + Other	10	285	7	24.56 (6.37–42.76)	Gastrointestinal: 4 Bone marrow suppression: 3
Tetracycline^c^ + Other	25	854	2	2.34 (0.00–5.59)	Gastrointestinal: 2
TMP-SMX + Other	22	609	13	21.35 (9.74–32.95)	Gastrointestinal: 4 Hyperkalemia: 3 Nephrotoxicity: 4 Allergy (rash): 1 Other: 1 (mouth sores)
**Total**	**119**	**3,755**	**37**	**9.85** **(6.68–13.03)**	Gastrointestinal: 20 Hyperkalemia: 4 Nephrotoxicity: 4 Bone marrow suppression: 3 Allergy (rash): 2 Joint pain: 1 Other: 3 (mouth sores, confusion)

a Levofloxacin or ciprofloxacin.

b Patients, days of therapy, and adverse events were most often counted in more than one category. For example, a patient receiving amoxicillin-clavulanate and levofloxacin was counted in the “Fluoroquinolone + Other” and “Beta-lactam + Other” categories.

c Doxycycline or minocycline.

Overall, linezolid and TMP/SMX had the highest ADE rates in both combination and monotherapy regimens. For linezolid, there were 20 total treatment courses; 10 (50.0%) of the 20 treatment courses resulted in at least one ADE. Four out of the 20 (20.0%) developed bone marrow suppression. The median number of days to develop bone marrow suppression was 13.5 (IQR: 10–17.5). All patients were discharged on the same dose of 600 mg twice a day. No therapeutic drug monitoring was used. Seven out of the 10 treatment courses that developed an ADE resulted in discontinuation of the drug; 2 of which were associated with readmission. For TMP/SMX, there were 62 total treatment courses; 19 (31%) of these treatment courses resulted in at least one ADE. Twelve out of the 62 patients (19.4%) developed nephrotoxicity and/or hyperkalemia. The median number of days to develop these adverse events was 11 (IQR: 4–20). Eight (66.7%) of the 12 patients who developed nephrotoxicity and/or hyperkalemia had diabetes. All patients were discharged on at least 2 double-strength (DS) tablets daily (320 mg of trimethoprim); however, most patients who developed an ADE were on at least 4 DS tablets daily. Ten patients were on 2 DS tablets daily of whom 4 had an ADE, 46 patients were on 4 DS tablets daily of whom 11 had an ADE, and 6 patients were on greater than 4 DS tablets daily of whom 4 had ADE). Sixteen of the 19 treatment courses who developed an ADE resulted in discontinuation of the drug; 3 of which were associated with readmission.

Lastly, very few patients were treated with oral cephalosporins, clindamycin, or metronidazole. The estimated ADE per 1,000 COpAT-days for these drug classes were poorly estimated given the low number of patient-days observed. There were no ADEs observed with clindamycin in 33 days of exposure. The ADE per 1,000 COpAT-days for cephalosporins and metronidazole are available upon request.

## Discussion

In this study evaluating the safety of prolonged courses of oral antibiotics, approximately one in four patients experienced an adverse event. Gastrointestinal adverse events were most common, but other adverse events were also observed. Combination regimens had more ADEs than monotherapy regimens although this was not statistically significant. Among combination regimens, linezolid, TMP/SMX, and fluoroquinolones had the highest ADE rate while combinations with tetracyclines and beta-lactams had lower ADE rates. Among monotherapy regimens, linezolid and TMP/SMX had the highest ADE rates, while fluoroquinolones and beta-lactams had lower ADE rates. In our analysis looking at both monotherapy and combination regimens, linezolid and TMP-SMX had the highest rates of adverse events while amoxicillin, amoxicillin-clavulanate, and tetracyclines had the lowest.

Overall, there are very few studies that have evaluated the safety profile of oral antibiotics used as COpAT. Sawey *et al.* published a study comparing ADE of patients receiving oral linezolid, TMP/SMX, voriconazole, and itraconazole under a COpAT monitoring protocol. Amongst patients who received linezolid, they also reported GI symptoms as the most common ADE. Regarding TMP/SMX, their study reported electrolyte and renal injury as the most common ADE followed by GI symptoms.^13^

In our study, 46 patients (26%) experienced 73 ADEs. This is overall higher compared to previous studies of ADE rates in OPAT patients. Keller *et al.* reported 18% of patients experienced an ADE in a prospective cohort study describing ADE rates in OPAT patients.^14^ However, OPAT in general introduces the risk for vascular access complications which is inherently avoided with oral therapy in COpAT patients. In an observational cohort study, Underwood *et al.* concluded catheter-related adverse events occur more frequently than drug-related adverse events.^15^ Therefore, while COpAT does not eliminate the risk for ADE, it still my offer an advantage of removing vascular access complication risk.

Our findings highlight several important considerations for clinicians when prescribing prolonged courses of oral antibiotics. Our rate of ADE reinforces the need for continued careful monitoring for COpAT patients. Although GI symptoms were the most frequent ADE across antibiotic classes, the type of ADEs varied substantially by antibiotic class suggesting that regimen choice should be individualized based on patients’ preexisting risk factors, medical problems, and even social factors that may affect laboratory monitoring and follow-up. For example, with linezolid, the occurrence of bone marrow suppression within a median of about two weeks suggests the need for early laboratory monitoring and potentially limiting to shorter treatment courses when alternatives exist. In regards to TMP/SMX, the rates of nephrotoxicity and hyperkalemia, particularly in patients with diabetes, suggests the need for closer laboratory monitoring and caution when used in patients with preexisting risk factors for renal toxicity. Most patients who developed an ADE with TMP/SMX were discharged on higher doses (at least 4 DS tablets daily). Therapeutic drug monitoring may also have a role in preventing ADEs for antibiotics with established exposure-toxicity relationships, such as linezolid.^16^

In contrast, oral beta-lactams and tetracyclines had lower ADE rates suggesting they are better tolerated for prolonged courses. However, this may be challenging in certain infections such as bone and joint infections as oral beta-lactams may be considered less effective given their poor bone penetration and bioavailability compared to other classes.^17,18^ Similarly, doxycycline typically achieves low serum concentrations compared to other classes such as linezolid or TMP/SMX, so is typically not used for patients with endovascular infections or bacteremia.^19^ Again, the need for an individualized approach for each patient is highlighted.

Fluoroquinolones were the third most prescribed antibiotic in our study. Gastrointestinal adverse events were the most common ADE in our study and serious complications were rare. Gastrointestinal symptoms are known to be a common side effect associated with fluoroquinolones; however, concerns about tendinopathy and cardiovascular risk remain prominent in the literature.^20^ There was only one report of joint pain in our study and no reports of cardiovascular complications from fluoroquinolone use, which may suggest more serious complications are less frequent than anticipated.

There were several limitations in our study, primarily its retrospective design and inability to attribute causality. In an effort to report clinically meaningful adverse events, we relied on clinician documentation of ADEs, which may be imprecise. If laboratory ADEs occurred but were not documented by the clinician, then these were not counted towards the incidence of ADEs. Similarly, we relied on documentation to determine when an acute kidney injury occurred as opposed to an expected rise in serum creatinine with TMP/SMX. Many patients were discharged on combinations of antibiotics making it difficult at times to ascertain the true ADE risk for each agent. We attempted to address this by providing analyses of ADEs for patients on monotherapy regimens. The number of ADEs observed was rather low and consequently our effect estimates have large CIs. Furthermore, although all patients had follow-up appointments scheduled in our infectious disease clinic, some patients did not attend their scheduled visit and were lost to follow-up. We still included these patients as we felt this is common in real-world clinical practice and frequently encountered. Regardless, this may lead to an underestimate of the incidence of ADEs. Additionally, we assumed a constant hazard throughout each day of antibiotic exposure, which may not be correct. This single center experience may also not apply to other centers with different patient populations or prescribing practices. Based on our patient cohort demographics, our results may not fully represent all patients discharged on oral therapy, especially in regard to an older patient population or patients with more comorbidities. Lastly, we only assessed one side of the risk-benefit equation and there may be many circumstances where more hazardous therapies are more likely to be effective.

In summary, most patients were able to tolerate prolonged courses of oral antibiotics with GI side effects as the most common ADE. Linezolid and TMP/SMX were found to have higher ADE rates, with GI symptoms, bone marrow suppression, nephrotoxicity, and hyperkalemia more commonly observed. Our findings suggest a few implications: first, clinicians should be judicious in selecting linezolid and TMP/SMX for prolonged courses and ensure adequate lab monitoring is in place. TMP/SMX may not be a preferred choice in patients with diabetes if other options are available. Second, the relatively favorable safety profile of oral beta-lactams and tetracyclines should be considered alongside their efficacy relative to other antibiotic classes depending on the infectious syndrome. Third, structured protocols for laboratory monitoring and therapeutic drug monitoring especially for higher risk patients such as those with diabetes or baseline renal impairment may be worth incorporating to mitigate risks associated with higher-toxicity regimens. Lastly, although the use of COpAT is rising to help mitigate potential harms associated with IV antibiotic use, the risk of ADEs associated with COpAT is still present; therefore, Antimicrobial Stewardship remains essential to guide antibiotic selection and duration. Our results emphasize the importance of weighing patient-specific risk factors and drug tolerability when planning prolonged oral antibiotic courses. However, more evidence-based studies that compare the efficacy and the safety profile of various oral antibiotics are needed to assist in the development of protocols for patients receiving COpAT.

## Supporting information

10.1017/ash.2026.10373.sm001 Siddique et al. supplementary material Siddique et al. supplementary material

## Data Availability

Deidentified data is available from the authors upon request.

## Supplementary material

The supplementary material for this article can be found at https://doi.org/10.1017/ash.2026.10373.
