# Supplementary material for: Pills and ills: adverse events associated with oral antibiotics for prolonged durations of COpAT treatment courses
Source: Antimicrob Steward Healthc Epidemiol. 2026 May 5;6(1):e123. doi: 10.1017/ash.2026.10373 (PMC13150467; doi:10.1017/ash.2026.10373)
Supplement: Siddique et al. supplementary material [file S2732494X26103738sup001.docx]

Supplementary Table 1. Specific Adverse Drug Events (ADE) Associated with Antibiotic Classes

| **Antibiotic Class and ADE Associated** | **Number of ADE** |
| --- | --- |
| ***Amoxicillin*** | **3** |
| Mouth Sores | 1 |
| GI symptoms | 2 |
|  |  |
| ***Amoxicillin/Clavulanate*** | **6** |
| GI symptoms | 5 |
| Rash | 1 |
|  |  |
| ***Azithromycin*** | **1** |
| GI symptoms | 1 |
|  |  |
| ***TMP/SMX*** | **26** |
| AKI | 11 |
| Hyperkalemia | 7 |
| GI symptoms | 5 |
| Rash | 2 |
| Mouth sores | 1 |
|  |  |
| ***Oral Cephalosporins*** | **1** |
| Confusion | 1 |
|  |  |
| ***Fluoroquinolones*** | **12** |
| GI symptoms | 9 |
| Body aches | 1 |
| Hyperkalemia | 1 |
| Joint pain | 1 |
|  |  |
| ***Tetracyclines*** | **4** |
| GI symptoms | 4 |
|  |  |
| ***Linezolid*** | **12** |
| Bone marrow suppression | 4 |
| GI symptoms | 6 |
| Tremor | 1 |
| Tongue soreness/color change | 1 |
|  |  |
| ***Metronidazole*** | **4** |
| GI symptoms | 1 |
| Joint pain | 1 |
| Tremor | 1 |
| Confusion | 1 |
|  |  |
| ***Rifampin*** | **4** |
| GI Symptoms | 4 |
|  | **Total: 73** |
